# Hepatic oncology diagnosis based on imaging fractal analysis: preliminary results

**DOI:** 10.1186/1878-5085-5-S1-A43

**Published:** 2014-02-11

**Authors:** Rostyslav V Bubnov, Ivan M Melnyk

**Affiliations:** 1 The Centre of ultrasound diagnostics and interventional sonography, Clinical hospital “Pheophania” of State Affairs Department, Kyiv, Ukraine; 2 International Research and Training Center for Information Technology and Systems NAS and MES of Ukraine, Kyiv, Ukraine

## Introduction

Biological and medical systems are predominantly irregular, complex and non-linear, since cannot be quantified by classical geometry approach. Novel mathematical algorithms can expand the information content of medical images, providing an objective measurement to reduce subjectivity in the perception and interpretation [1,2]. The aim of the study was to assess the capabilities of fractal analysis for expand its diagnostic value of diagnostic imaging.

## Methods

Fractal Dimension (FD) is a statistical quantity that gives an indication of how completely a fractal appears to fill space, zooming down to more finer scales. We proposed a method of medical images analysis obtained from a wide range of sources - radiology imaging. The fractal parameters of these images were calculated for 7 patients with liver lesions for ultrasound, CT, MRI images and generated 3D vector and voxel models by patented method, "covering" the parts of these expertly segmented images by two-dimensional geometric shapes (squares, rectangles, triangles, circles, ellipses) and three-dimensional (cubes, simplices, balls, ellipsoids, pyramids) with applying iteration method, which involves finding the appropriate (i-th) value approaching the value of FD.

## Results

FD was estimated as 1.67 for hepatocellular carcinoma case; 1.72 - for cholangiocarcinoma; 1.45-1.56 for complex cysts; and 1.15-1.35 for metastases. We consider that only three-dimensional reconstruction from expertly segmented images allows to perform accurate analysis. The most informative description of self-similarity is fractal analysis, conducted with the maximum number of steps. However, objective analysis is limited by resolution of diagnostic equipment, is possible only under visual control by expert. The application of automated and semi-automated image analysis leads to control the process, correctly selecting the areas for research, preselecting a suppositive fractal structure.

## Conclusion

Fractal analysis of medical images is a promising non-invasive sophisticated approach, it should become an highly informative indicator of pathological formations using nonlinear mathematical parameters of structure, gives insights into tumor morphology and can become a useful tool for analyzing tumor growth patterns for diagnosis, staging and treatment follow up.

## Recommendations

Further studies on large patient cohorts are recommended to assess different pathological processes to establish scientifically valid standards.
